# Supplementary material for: Lactobacillus paragasseri LPG-9 reduces placental inflammation in intrahepatic cholestasis of pregnancy by regulating TGR5 in mice
Source: Commun Biol. 2026 Mar 26;9:679. doi: 10.1038/s42003-026-09869-4 (PMC13187033; doi:10.1038/s42003-026-09869-4)
Supplement: Supplementary file 2 — Supplementary information [file 42003_2026_9869_MOESM2_ESM.pdf]

Supplementary materials for

Lactobacillus paragasseri LPG-9 Reduces Placental Inflammation in Intrahepatic Cholestasis of Pregnancy by regulating TGR5 in mice

Wanwen Huang[1], Jiechang Zhang[2], Jiamin Shan[2], Wei Shen[3], Pingping Du[4], Jiaxin Liu[2], Xiaotong Guo[2], Zhenhui Chen[2], Weisen Zeng[5]*, Qiongxi Lin[2]* and Hongying Fan[2]*

1 Experimental Teaching Center of Preventive Medicine, Guangdong Provincial Key Laboratory of Tropical Disease, School of Public Health, Southern Medical University, Guangzhou, China

2 Department of Microbiology, Guangdong Provincial Key Laboratory of Tropical Disease Research, School of Public Health, Southern Medical University, Guangzhou, China

3 Department of Neonatology, Nanfang Hospital, Southern Medical University, Guangzhou, China

4 Department of Obstetrics and Gynaecology, Nanfang Hospital, Southern Medical University, Guangzhou, China

5 Department of Cell Biology, School of Basic Medical Science, Southern Medical University, Guangzhou, China.

*Address correspondence to: Hongying Fan, gzfhy@smu.edu.cn; Qiongxi Lin, lqx123@smu.edu.cn; and Weisen Zeng, zengws@smu.edu.cn.

Postal address: No.1023-1063, Shatai South Road, Baiyun District, Guangzhou, China

Supplementary information

Supplementary Figure 1. Pathological features of humans and mice with ICP and validation of molecular interactions.
Supplementary Figure 2. Immunohistochemical analyses in all five ICP patients and five healthy controls.
Supplementary Figure 3. Activation of TGR5 improves ICP-associated placental inflammation.
Supplementary Figure 4. Gut microbiota intervention affects liver tissue pathology and placental neutrophil infiltration in ICP.
Supplementary Figure 5. Agarose gel electrophoresis for assessing RNA integrity
Supplementary Figure 6. The original protein blotting pattern image.
Supplementary Table 1. Primers used in this study.

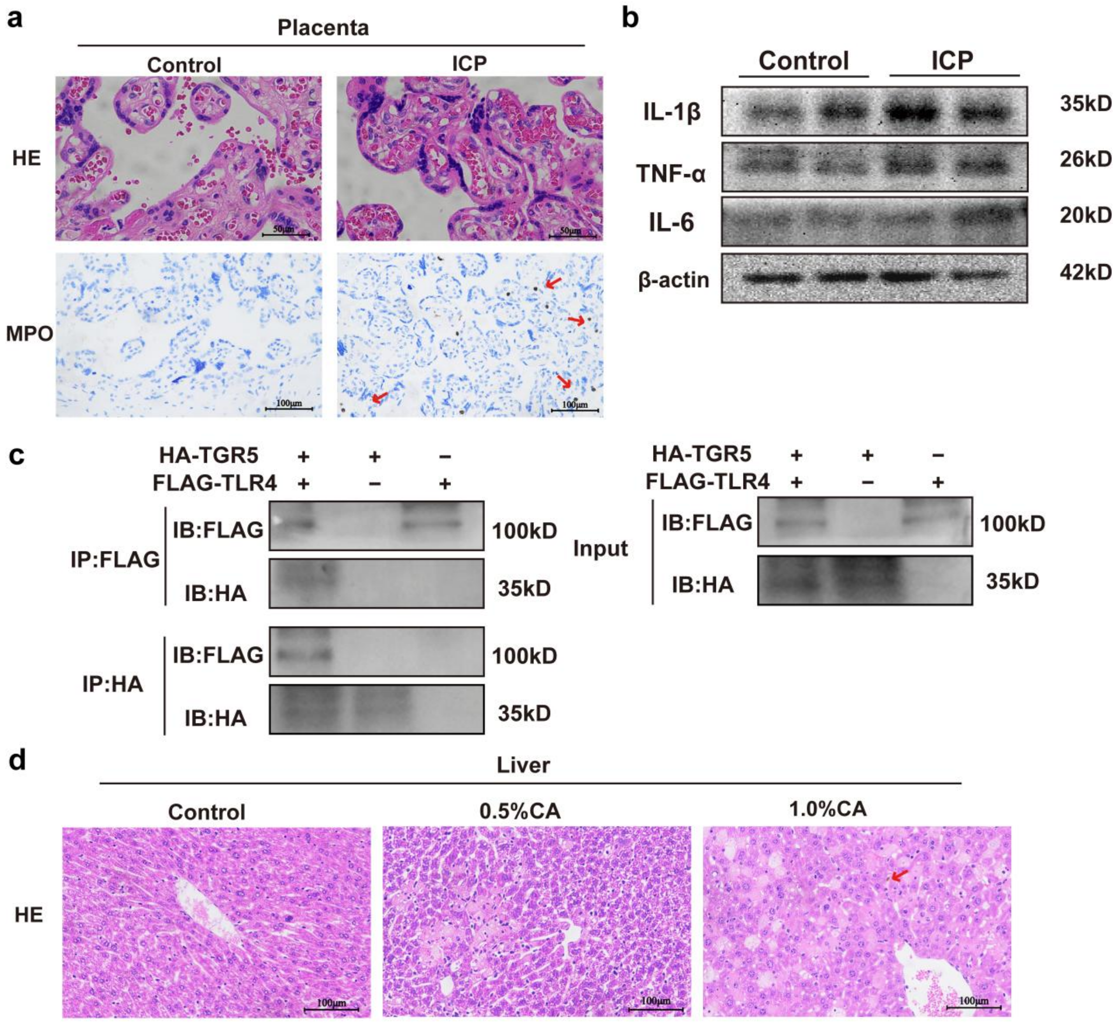

Supplementary Figure 1. Pathological features of humans and mice with ICP and validation of molecular interactions

a. Increased neutrophil infiltration in the placental tissues of patients with ICP (400×, HE； 200×, MPO). Arrows indicate neutrophils; b. Western blotting of pro-inflammatory cytokines in the human placental tissue; c. Co-immunoprecipitation of TGR5 and TLR4 protein in *HEK293T* cells; d. Hematoxylin-eosin-stained sections of the liver in the ICP model mice, 200×. Arrows indicate bile plugs.

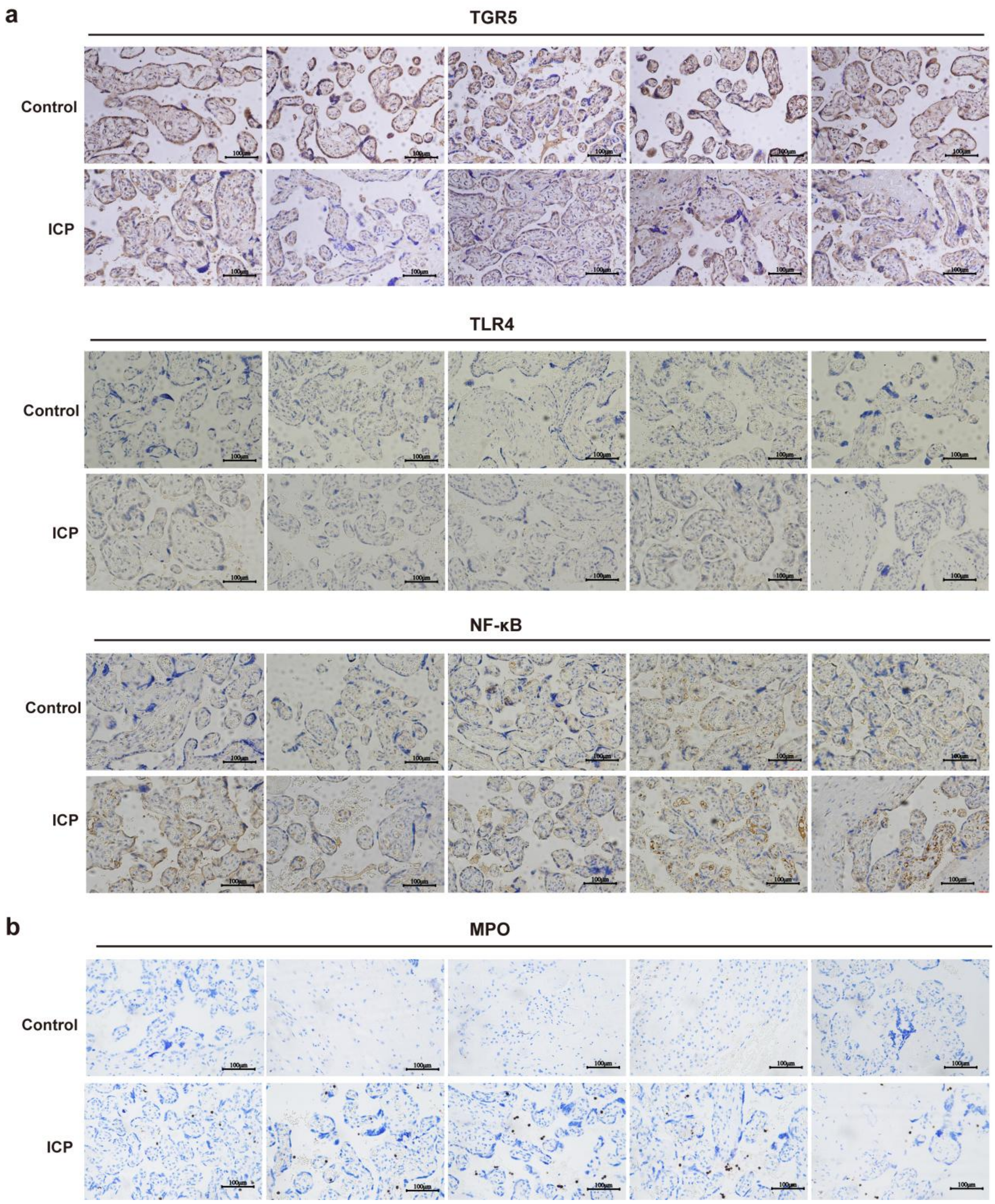

Supplementary Figure 2. Immunohistochemical analyses in all five ICP patients and five healthy controls.

a. Immunohistochemical analysis of TGR5, TLR4 and phospho-NF-κB p65 in the placental tissues of patients with ICP, 200×; b. Increased neutrophil infiltration in the placental tissues of patients with ICP (200×, MPO).

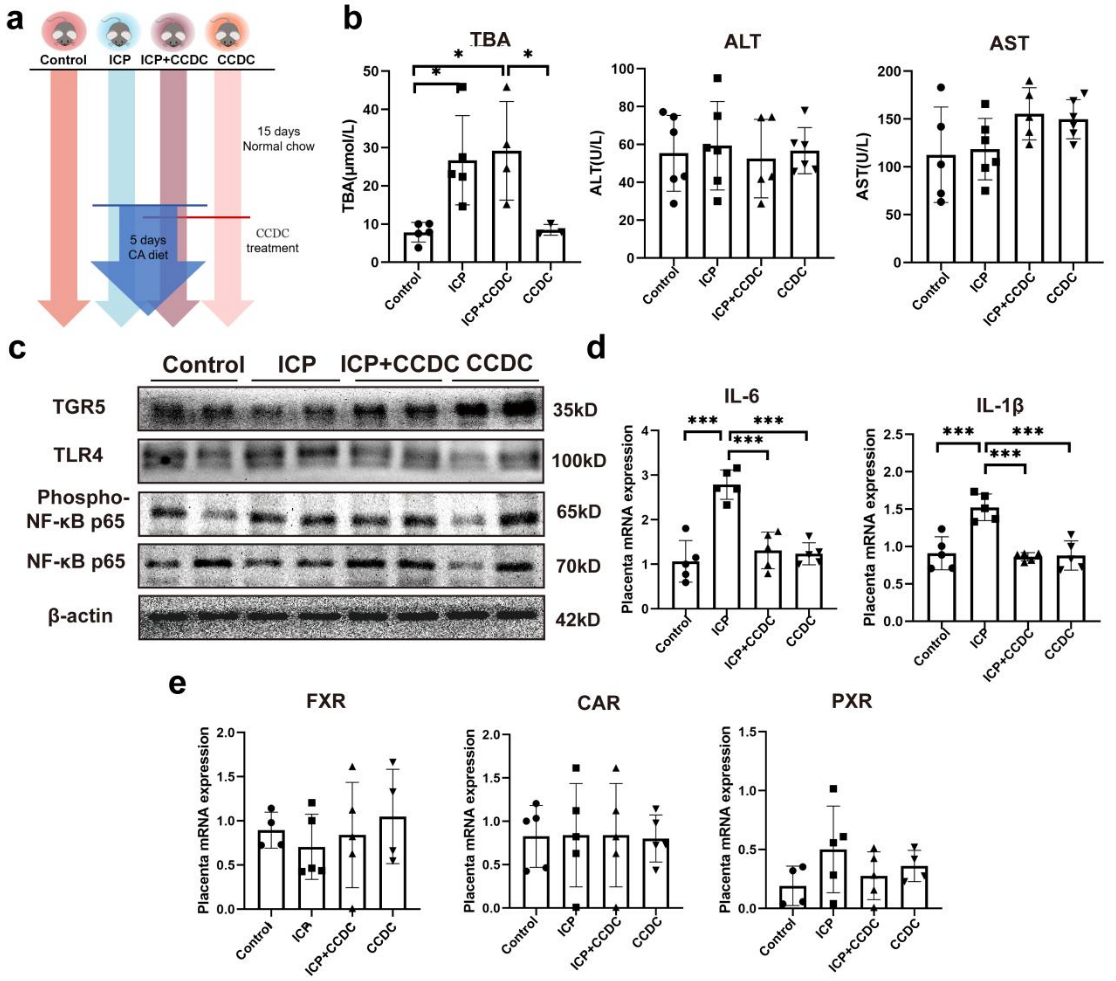

Supplementary Figure 3. Activation of TGR5 improves ICP-associated placental inflammation

A. Schematic representation of CCDC treatment; b. Serum TBA, AST and ALT levels in the ICP model mice treated with a TGR5 receptor agonist (CCDC) (*n* = 5-6 biological replicates; one-way ANOVA); c. Western blotting of placental TGR5, TLR4 and NF-κB; d. Quantitiative polymerase chain reaction (qPCR) data showing the gene expression levels of placental pro-inflammatory cytokines (*n* = 5-6 biological replicates; one-way ANOVA); e.mRNA expression levels of FXR, PXR, and CAR after CCDC intervention. Error bars represent standard deviation. Error bars represent standard deviation. Significance levels: *p <0.05, **p <0.01, ***p <0.001.

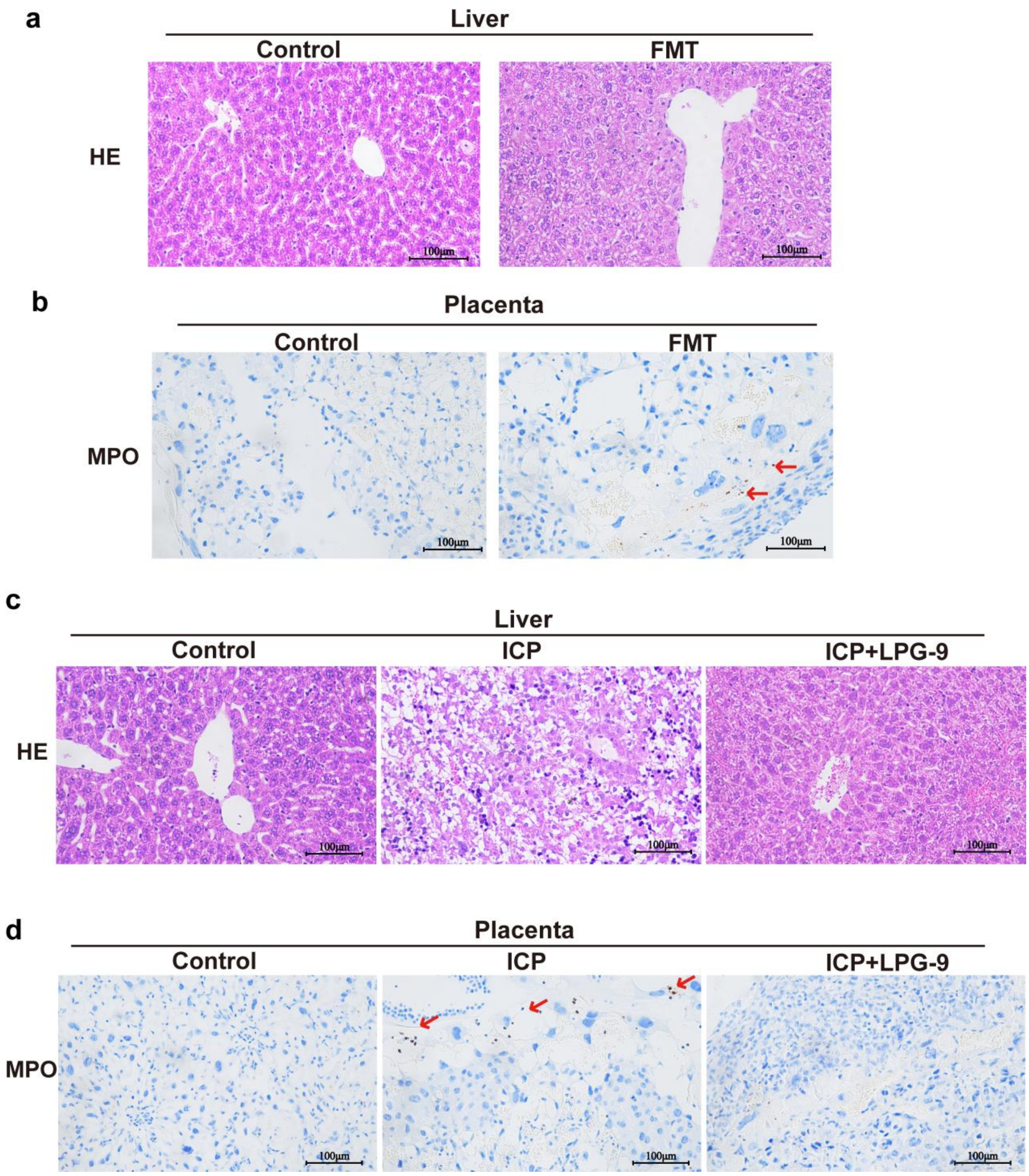

Supplementary Figure 4. Gut microbiota intervention affects liver tissue pathology and placental neutrophil infiltration in ICP

a. Hematoxylin-eosin-stained sections of the liver after FMT treatment, 200×; b. Myeloperoxidase-stained sections of the placenta after FMT treatment, 200×. Arrows indicate neutrophils; c. Hematoxylin-eosin-stained sections of the liver after LPG-9 treatment, 200×; d. Myeloperoxidase-stained sections of the placenta after LPG-9 treatment, 200×. Arrows indicate neutrophils.

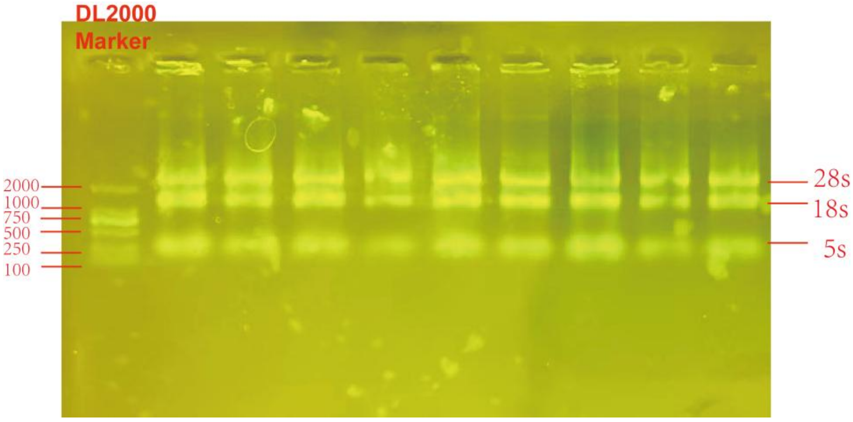

Supplementary Figure 5. Agarose gel electrophoresis for assessing RNA integrity

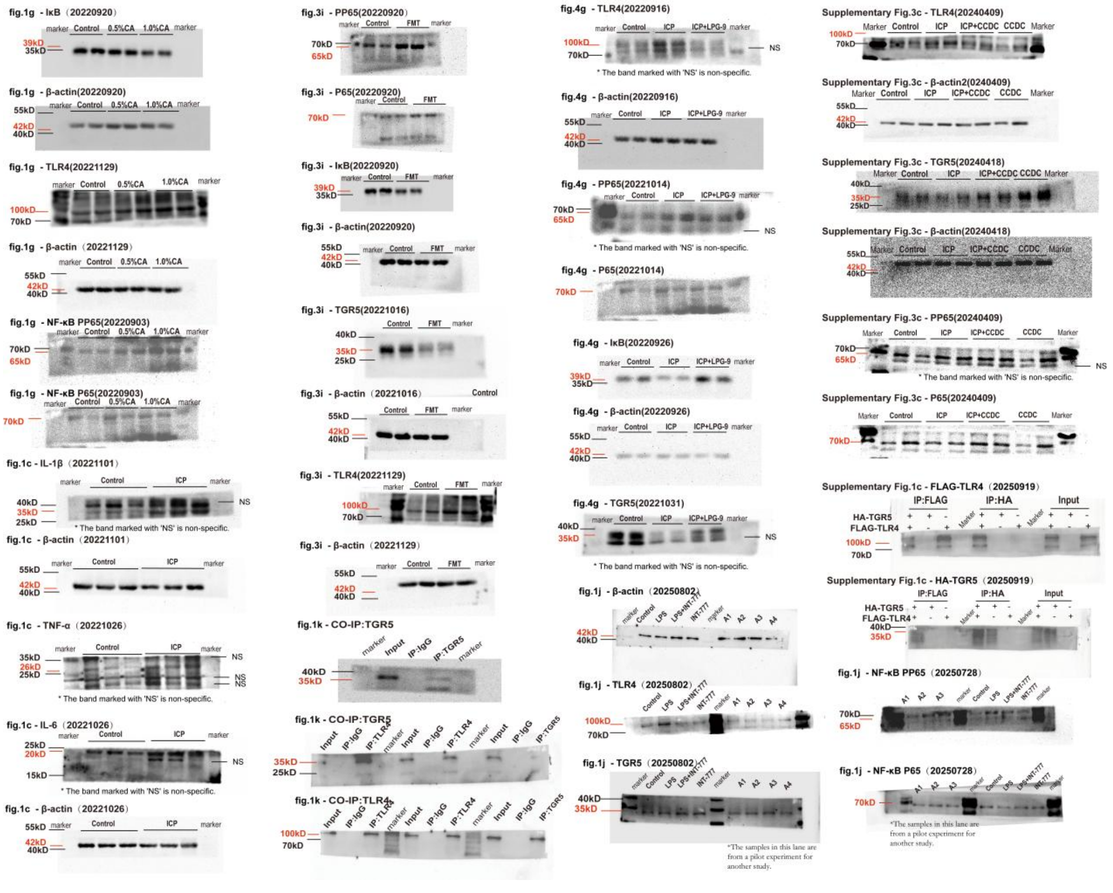

Supplementary Figure 6. The original protein blotting pattern image.

For each target protein, the band corresponding to the expected molecular weight are labeled in red. Nonspecific bands visible in some lanes are labeled "NS". These bands likely result from the polyclonal nature of the antibodies and the complexity of placental tissue lysates, and do not represent the target protein. Molecular weight markers (kDa) are indicated.

Supplementary Table 1. Primers used in this study

Gene		Primer sequence
GAPDH	Forward	AGCTTGTCATCAACGGGAAG
	Reverse	TTTGATGTTAGTGGGGTCTCG
NF-κB	Forward	CCTCTGGCGAATGGCTTTACT
	Reverse	GCTGGCTCTGAGGGAAAGATG
TGR5	Forward	GTCCTGCCTCCTTCTCCACT
	Reverse	CCAGGTGAGGAACAGGGCTA
IL-1β	Forward	GAAGTTGACGGACCCCAAAA
	Reverse	CCACAGCCACAATGAGTGATAC
TNF-α	Forward	TCGTAGCAAACCACCAAGTG
	Reverse	GGAGTAGACAAGGTACAACCCA
IL-6	Forward	AGTTGCCTTCTTGGGACTGA
	Reverse	TCCACGATTTCCCAGAGAAC
Ntcp	Forward	AGGGGGACATGAACCTCAG
	Reverse	TCCGTCGTAGATTCCTTTGC
Bsep	Forward	CCAGAACATGACAAACGGAA
	Reverse	AAGGACAGCCACACCAACTC
OSTα	Forward	CAGCGTCTGCCTGAGAGAAA
	Reverse	GGTGAGGGCTATGTCCACTG
OSTβ	Forward	GAAACATGGACCACAGTGCAG
	Reverse	GCCAGGACCAGGATGGAATAA
Asbt	Forward	CTTCTCCCCCGAGGATCTCA
	Reverse	TGATGGCCTGGAGTCCATTTC
OATP	Forward	CCAGGCCCATAACCACACATC
	Reverse	GTGCATACCTAGCCAAATCACT
bsh	Forward	CACATATTGTGGCACGAACAATHGARTGGGG
	Reverse	CTGTGCCCGGATACAGATTAACRTARTTRTT
baiCD	Forward	GGWTTCAGCCCRCAGATGTTCTTTG
	Reverse	GAATTCCGGGTTCATGAACATTCTKCKAAG
16sRNA	Forward	GTGSTGCAYGGYTGTCGTCA
	Reverse	ACGTCRTCCMCACCTTCCTC
FXR	Forward	CTTGATGTGCTACAAAAGCTGTG
	Reverse	ACTCTCCAAGACATCAGCATCTC
16S RNA	338F	ACTCCTACGGGAGGCAGCAG
	806R	GGACTACHVGGGTWTCTAAT
