# Supplementary material for: Lactobacillus paragasseri LPG-9 reduces placental inflammation in intrahepatic cholestasis of pregnancy by regulating TGR5 in mice
Source: Commun Biol. 2026 Mar 26;9:679. doi: 10.1038/s42003-026-09869-4 (PMC13187033; doi:10.1038/s42003-026-09869-4)
Supplement: Supplementary file 3 — Description of Additional Supplementary Files [file 42003_2026_9869_MOESM3_ESM.pdf]

Description of Additional Supplementary Files

File name: Supplementary Data 1
Description: Original data of the charts in the article.
