# Supplementary material for: Lactobacillus paragasseri LPG-9 reduces placental inflammation in intrahepatic cholestasis of pregnancy by regulating TGR5 in mice
Source: Commun Biol. 2026 Mar 26;9:679. doi: 10.1038/s42003-026-09869-4 (PMC13187033; doi:10.1038/s42003-026-09869-4)
Supplement: Supplementary file 5 — Summary [file 42003_2026_9869_MOESM5_ESM.pdf]

nature portfolio

Corresponding author(s): Hongying, Fan

Last updated by author(s): Feb 25, 2026

Reporting Summary

Nature Portfolio wishes to improve the reproducibility of the work that we publish. This form provides structure for consistency and transparency in reporting. For further information on Nature Portfolio policies, see our Editorial Policies and the Editorial Policy Checklist.

Statistics

For all statistical analyses, confirm that the following items are present in the figure legend, table legend, main text, or Methods section.

n/a	Confirmed	
☐	☒	The exact sample size (n) for each experimental group/condition, given as a discrete number and unit of measurement
☐	☒	A statement on whether measurements were taken from distinct samples or whether the same sample was measured repeatedly
☐	☒	The statistical test(s) used AND whether they are one- or two-sided<br>*Only common tests should be described solely by name; describe more complex techniques in the Methods section.*
☐	☒	A description of all covariates tested
☐	☒	A description of any assumptions or corrections, such as tests of normality and adjustment for multiple comparisons
☐	☒	A full description of the statistical parameters including central tendency (e.g. means) or other basic estimates (e.g. regression coefficient) AND variation (e.g. standard deviation) or associated estimates of uncertainty (e.g. confidence intervals)
☐	☒	For null hypothesis testing, the test statistic (e.g. F, t, r) with confidence intervals, effect sizes, degrees of freedom and P value noted<br>*Give P values as exact values whenever suitable.*
☐	☒	For Bayesian analysis, information on the choice of priors and Markov chain Monte Carlo settings
☐	☒	For hierarchical and complex designs, identification of the appropriate level for tests and full reporting of outcomes
☐	☒	Estimates of effect sizes (e.g. Cohen's d, Pearson's r), indicating how they were calculated

Our web collection on statistics for biologists contains articles on many of the points above.

Software and code

Policy information about availability of computer code

Data collection	IBM SPSS was used for data collection.
Data analysis	IBM SPSS, GraphPad Prism and R were used for statistical analysis.

For manuscripts utilizing custom algorithms or software that are central to the research but not yet described in published literature, software must be made available to editors and reviewers. We strongly encourage code deposition in a community repository (e.g. GitHub). See the Nature Portfolio guidelines for submitting code & software for further information.

Data

Policy information about availability of data

All manuscripts must include a data availability statement. This statement should provide the following information, where applicable:

- Accession codes, unique identifiers, or web links for publicly available datasets
- A description of any restrictions on data availability
- For clinical datasets or third party data, please ensure that the statement adheres to our policy

Provide your data availability statement here.

Research involving human participants, their data, or biological material

Policy information about studies with human participants or human data. See also policy information about sex, gender (identity/presentation), and sexual orientation and race, ethnicity and racism.

Reporting on sex and gender	The samples uesd in the study were placental samples, so we were not classified by gender or sex.
Reporting on race, ethnicity, or other socially relevant groupings	This study dose not cover this topic.
Population characteristics	This study dose not cover this topic.
Recruitment	All participants voluntarily participated.
Ethics oversight	NFEC-2021-054

Note that full information on the approval of the study protocol must also be provided in the manuscript.

Field-specific reporting

Please select the one below that is the best fit for your research. If you are not sure, read the appropriate sections before making your selection.

☒ Life sciences ☐ Behavioural & social sciences ☐ Ecological, evolutionary & environmental sciences

For a reference copy of the document with all sections, see nature.com/documents/nr-reporting-summary-flat.pdf

Life sciences study design

All studies must disclose on these points even when the disclosure is negative.

Sample size	The sample size was calculated using the resource equation medthod, combined with a priori using a power analysis.
Data exclusions	The excluded data and the reasons for exclusion are all described in article.
Replication	All the repeated exprienments were successful.
Randomization	Mice were randomly assigned to groups according to gestational order.
Blinding	Throughout the experimenr, researchers reponsible for tissue collection and histological analysis remained blinded to group assignments.

Reporting for specific materials, systems and methods

We require information from authors about some types of materials, experimental systems and methods used in many studies. Here, indicate whether each material, system or method listed is relevant to your study. If you are not sure if a list item applies to your research, read the appropriate section before selecting a response.

Materials & experimental systems

n/a	Involved in the study	
☐	☒	Antibodies
☐	☒	Eukaryotic cell lines
☒	☐	Palaeontology and archaeology
☐	☒	Animals and other organisms
☒	☐	Clinical data
☒	☐	Dual use research of concern
☒	☐	Plants

Methods

n/a	Involved in the study	
☒	☐	ChIP-seq
☒	☐	Flow cytometry
☒	☐	MRI-based neuroimaging

Antibodies

Antibodies used	Anti-TLR4 mouse monoclonal(66350,Proteintech), anti-TGR5 rabbit(A20778, ABclonal, China),anti-phospho-NF-κB P65 rabbit(3033S,Cell Signaling),anti-NF-κB rabbit(AB32536, ABcam), anti-HA(R20003, Abmart), anti-FLAG(AF519, Beyotime) antibodies.
Validation	Mouse monoclaonal anti-TLR4, which recognizes human, mouse, rat, and porcine TLR4 and has been validated for WB, IHC, and ELISA

Validation	according to the manufacturer's datasheet; rabbit anti-TGR5, validated for WB and IF/ICC with reactivity to human and rat, showing a specific band at 35 kDa in rat samples; rabbit monoclonal anti-phospho-NF-κB p65, which specifically detects endogenous levels of p65 only when phosphorylated at Ser536 and shous reactivity with human, mouse, rat, hamster, monkey, and pig; rabbit monoclonal anti-NF-κB p65, which has been validated by knockout studies, reacts with human and mouse, and is suitable for WB, IHC-P, ICC/IF, and IP applications.

Eukaryotic cell lines

Policy information about cell lines and Sex and Gender in Research

Cell line source(s)	Mouse macrophage J774.2 cell.
Authentication	The cell line has been verified.
Mycoplasma contamination	The detection of mycoplasma infection in the cell line was negative.
Commonly misidentified lines (See ICLAC register)	None.

Animals and other research organisms

Policy information about studies involving animals; ARRIVE guidelines recommended for reporting animal research, and Sex and Gender in Research

Laboratory animals	8-12 weeks old C57BL/6 mice.
Wild animals	The research dose not involve wild animals.
Reporting on sex	The study only used female mice.
Field-collected samples	The research dose not involve wild animals.
Ethics oversight	The Ethics Committee of Southern Medical University(permit NO.4400210006397)

Note that full information on the approval of the study protocol must also be provided in the manuscript.

Plants

Seed stocks	NO.
Novel plant genotypes	NO.
Authentication	NO.
